# Dear Readers and Authors

**DOI:** 10.34763/jmotherandchild.20202404.edit.04_2020

**Published:** 2021-07-16

**Authors:** Aleksandra Jezela-Stanek

**Affiliations:** 1 Department of Genetics and Clinical Immunology; National Institute of Tuberculosis and Lung Diseases; Warsaw; Poland

The editors of the Journal of Mother and Child/Medycyna Wieku Rozwojowego invite you to read the fourth issue of our quarterly. The topics that are discussed in this issue encompass congenital malformations, including an excellent institutional review on over ten years’ experience with H-type tracheooesophageal fistula (H-TEF) and a case study on management with wrap disruption after Nissen fundoplication in a child with gastro-oesophageal reflux after congenital oesophageal atresia; perinatology comprising diagnostic challenges in a patient with neonatal cholestasis and growth restriction in twin pregnancies; and finally, an original paper presenting an association between single nucleotide polymorphisms (SNPs) and viral load (VL) in congenital cytomegalovirus infection.

We start reading with an original article describing in detail 18 patients with a rare condition – congenital H-TEF – who were managed for over 10 years in the department of Paediatric Surgery at a tertiary institute (Tiwari C et al.). Tracheoesophageal fistula (TEF) without associated oesophagal atresia (EA), commonly called H-type or N-type or isolated fistula, is a rare congenital anomaly that accounts for 4–5% of all occurrences of congenital tracheoesophageal malformation. The malformation must be considered in the differential diagnosis when managing the patients with recurrent lower respiratory tract infection and in patients with ‘coughing and choking episodes. The repair of an H-type TEF is not a simple procedure and should not be taken lightly, since it is known to be associated with complications, i.e. damage to the recurrent laryngeal nerve.

We will continue with another excellent original paper by Jedlińska-Pijanowska and colleagues, who try to define the role and significance of SNPs, which may determine VL in congenital CMV (cCMV) infection. The authors concentrated on eight polymorphisms in genes encoding cytokines or cytokine receptors (*IL1B* rs16944, *IL12B* rs3212227, *IL28B* SNPs rs12979860, *CCL2* rs1024611, *DC-SIGN* rs735240, *TLR2* rs5743708, *TLR4* rs4986791 and *TLR9* rs352140) which were analysed in a study population of 233 newborns. They found *IL12B* rs3212227 to be associated with VL in cCMV, and symptomatic newborns had significantly higher viremia and viruria. Thus, we believe that SNPs’ role in the pathogenesis of cCMV is a clinically interesting subject and warrants further investigations.

Is growth restriction in twin pregnancies a double challenge? The answer to that question was the third article’s subject in this issue (Filipecka-Tyczka et al.). Foetal growth restriction is one of the most frequent prenatal pathologies, which complicates about 25–47% of twin pregnancies (vs. only 8% of singletons). A literature search was performed to present a comprehensive, critical and objective analysis of the current knowledge on growth restriction in twin pregnancies, including PubMed and SCOPUS, based on Medical Subject Headings (MeSH) terms.

Professor Jaak Jaeken sensitises us that in a patient with a complex pathology, the possibility that he/she is affected by several genetic/metabolic diseases should also be considered. This is certainly not a common situation, but it is undoubtedly a diagnostic challenge. We encourage you to read the attention-warranting description of the patient, who was finally found to suffer from three rare diseases.

The issue is closed with a mini-review on gastro-oesophageal reflux management after congenital oesophageal atresia (Dybowska et al.). In about 86% of cases, EA is accompanied by a lower TEF. Although corrective surgery restoring oesophageal continuity is carried out in the first days of a child’s life, patients may be required to undergo life-long complications associated with a congenital anomaly that are possibly the consequences of surgical treatment. As stated by the authors, ‘In the case of relapses of reflux oesophagitis in patients after anti-reflux surgery, the possibility of distant complications such as dislocation or dissolution of the fundoplication cuff should be taken into consideration’; this is their take-home message.

We hope that the presented papers will meet your expectations and encourage you to read the further issues of our Journal.

